# Management of maxillary polyposis through siddha - A case report

**DOI:** 10.6026/973206300213374

**Published:** 2025-09-30

**Authors:** Shanmugapriya P., Gayatri R., Varshini K., Subathra T., Ramamurthy Murugan

**Affiliations:** 1 Department of Nanju Maruthuvam, National Institute of Siddha, Affiliated to the Tamil Nadu Dr. MGR Medical University, Tambaram sanatorium, Chennai - 47, Tamil Nadu, India; 2 Department of Noi Naadal, National Institute of Siddha, Affiliated to the Tamil Nadu Dr. MGR Medical University, Tambaram Sanatorium, Chennai-47, Tamil Nadu, India; 3 Domain Expert, National Institute of Siddha, Affiliated to the Tamil Nadu Dr. MGR Medical University, Tambaram Sanatorium, Chennai-47, Tamil Nadu, India

**Keywords:** Inflammatory polyp, antrochoanal polyp, functional endoscopic sinus surgery, siddha

## Abstract

A 45-year-old male with a 7-years history of recurrent antrochoanal polyp (ACP) and severe left nasal epistaxis sought Siddha treatment after conventional therapies failed. He was treated for 14 months with *Impooral ilagam*, Vasantha kusumakara mathirai, Adathodai manappagu, *Seenthil chooranam* and *Muthu parpam*, including drug holidays. Nasal bleeding reduced from daily episodes to once every two months and eventually ceased, with no recurrence during follow-up. This case suggests Siddha medicine may offer a promising non-invasive alternative for managing chronic ACP, warranting further investigation in larger studies.

## Background:

According to the International classification of diseases, 11th revision, Antrochoanal polyps (ACP) fall under nasal polyp subgroups, which are defined as an inflammatory mass with a proliferating nature that arises from the nasal and paranasal mucosal linings which has a grayish white color and glutinous, agar-like masses with smooth surfaces [1]. Prevalence of antrochoanal polyps in the general population is only 4-6% of all nasal polyps [2]. Presentation of ACPs is mostly unilateral with a chief feature of nasal obstruction and rhinorrhea [3]. However, in some cases, rare manifestations like nasal bleeding had also been observed [4]. It affects any age group between 5- 81 years with the mean age of 40 years [5]. The conventional treatment employed for this condition is Functional Endoscopic Sinus Surgery (FESS) for the complete removal of polyp and restoration of sinus drainage system [6, 7]. Even though it is considered as standard of care for nasal polyp, this technique has few major complications such as cerebrospinal fluid leak, meningitis, hemorrhage and orbital injuries [8]. Even after FESS procedure, the recurrence rate was found to be 72.9 percent [9] which leads to the revision surgery which was considered to have a high risk of complications due to the distorted anatomy and scarring [10]. In Siddha, nasal polyps are described as *mulai mookkadaippu*, which are one among 9 types of *Mookkadaippu Noigal* according to *Yugi Vaidya Sinthamani*, [11] mainly caused by dust, polluted air, cold water bathing, consuming cold foods.

In Siddha, various treatment modalities exist for addressing nasal polyps which include external medications such as *nasiyam*, *naasikaparanam*, *pottanam*, *pugai*, *vedhu* and here, the case report deals with the ACP failed by various allopathic treatment strategies such as steroidal sprays, FESS and other AYUSH conventional therapies; treated by *Impooral ilagam*, *Vasantha kusumakara mathirai (VKM)*, *Adathodaimanappagu*, *Seenthil chooranam* and *Muthu parpam* [12]. The drugs chosen are based on the Siddha principles of '*mukkutram*.' Adathodai manappagu and VKM are scientifically proven drugs for the management of respiratory-related diseases, particularly COVID-19 and dengue [13, 14-15]. *Seenthil chooranam*, impooral and *Muthu parpam* show potent antioxidant and anti-inflammatory activity, which may help act against inflammatory polyposis [16, 17-18]. All of these medicines are approved by the Drug and Cosmetic Act, The Siddha Formulary of India, Part I, published by the Department of Health and Family Welfare, Government of India [19]. Therefore, it is of interest to describe the case of an antrochoanal polyp treated with this selected Siddha regimen.

## Patient information:

A 45-year-old male patient, working as a sales executive, presented to National institute of Siddha OPD, with the primary concern of recurrent bleeding from left nostril that even made his handkerchief become moistened with blood. Haemorrhage intensified, particularly following the lowering of head or after sneezing for 15 days. In addition, he had severe left nasal blockage, sneezing, rhinorrhea. He already had history of Hypothyroidism and Dyslipidemia since 2016 and had been under regular allopathic medications for that. While general examination, there was no pallor, icterus, clubbing of nails, oedema or lymphadenopathy noted. No Central Nervous System pathology was noted on thorough clinical examination. No relevant family history and personal history was identified. Patient did not have any sort of addictions and having a non-vegetarian dietary habit. Seven years ago, he had the similar symptoms of left nasal epistaxis, nasal block and underwent treatment with antibiotics, NSAIDs and a steroidal nasal spray and even went through other AYUSH treatments initially, providing only temporary relief. Subsequently, he underwent Functional Endoscopic Sinus Surgery (FESS) and the excised tissue was subjected to histopathological examination, revealing an inflammatory polyp as detailed in image 1. After 4 years, epistaxis, nasal blockage and rhinorrhoea recurred, he went for other conservative AYUSH management for the period of 6 months but it leaded to severe nasal bleeding and during the course, gallbladder's stone were detected for which he underwent surgical removal of the stones. Then he went through a second FESS procedure and the excised tissue again confirmed as left maxillary inflammatory polyp as detailed in image 2. However, 45 days after the latest surgery, the patient was experiencing similar symptoms but reluctant to undergo another surgical intervention. Consequently, he had sought Siddha management for his present condition.

## Clinical findings:

## Physical examination:

[1] Inspection - Round, Glossy, soft, greyish swelling in the middle meatus was seen with mild bleeding.

[2] Palpation - Nothing specific

[3] Examination of Paranasal Sinus - Maxillary Sinus tenderness Present

## Investigations:

While performing routine haematological and urine investigations it was found out none of the findings were pathologically significant to the diagnosis. PNS CT Scan as detailed in image 3 and Histopathological studies brought by the patient which helped in confirming diagnosis which was taken on 2016 and 2021 respectively. First CT PNS suggested heterogeneously enhancing lesion in the left maxillary sinus was likely to be neoplastic in aetiology. So, the patient underwent a histopathological study to confirm the diagnosis. Histopathology study revealed predominantly necrotic fibro collagenous tissue bits focally lined by respiratory and squamous epithelium. Neutrophilic exudates which helped to confirm diagnosis as inflammatory polyp mixed with karyorrhectic debris and large areas of haemorrhage are seen too. No fungal hyphae or spores were seen. Hence diagnosis was confirmed as: Inflammatory polyps. Histopathology of excised growth from left maxillary sinus section showed mostly necrosed tissue with inflammatory tissue. Abundant myxomatous tissue with ciliated columnar epithelial lining is seen. These epithelial cells are seen with densely infiltration of chronic inflammatory cells in the stroma. Large number of lymphocytes, histocytes and macrophages were seen. Myxomatous tissue is seen underneath. Massive stromal oedema and mild inflammatory reactions is also associated. All were mature and well differentiated. No suspicious area for malignancy seen. Figure 1 (see PDF) depicts the timeline of symptoms and management given for the patient.

## Diagnostic assessment:

Histopathology study reveals predominantly necrotic fibro collagenous tissue bits focally lined by respiratory and squamous epithelium. Neutrophilic exudate mixed with karyorrhectic debris and large areas of haemorrhage were seen too. No fungal hyphae or spores were seen hence fungal sinusitis could be differentiated. Plain CT scan of Para Nasal Sinus study revealed mucosal thickening with soft tissue density was seen filling left maxillary sinus, left adjacent nasal cavity, reaching up to both external and internal choana with attenuation of nasal air passage. Mild mucosal thickening was also seen in bilateral ethmoidal and right maxillary sinuses.

## Diagnostic reasoning:

Histopathological examination plays a crucial role in diagnosing and differentiating ACP from other vascular lesions [20]. It also differentiates there is no evidence for fungal sinusitis and any other malignant type of lesions.

## Prognostic characteristics:

There was no recurrence or malignant transformation of the condition, which shows a good prognosis [21].

## Therapeutic interventions:

Table 1 (see PDF) depicts the types of therapeutic interventions used.

## List of Abbreviations:

## Abbreviation definition:

ACP: Antrochoanal Polyp

VKM: Vasantha Kusumakara Maathirai

FESS: Functional Endoscopic Sinus Surgery

HPE: Histo Pathological Examination

## Drug holidays and Pathiyam:

Medications were administered with the appropriate drug holidays, which were seven days of medication and two days of rest. Patient was advised to take oil bath twice a week with *Chukku thailam*. The pathiyam (treatment diet) mentioned for Kabam diseases was adhered during the entire course since mookkadaippu noi falls under that. The diet free from sour taste, spiciness, food with cold potency, cold water, vegetables like pumpkin, ridge gourd, bottle gourd, snake gourd were avoided. Moreover, sprouts, green leafy vegetables, butter milk were consumed. The patient was advised to follow Pathiyam diet as a routine.

## Follow up and outcomes:

## Clinician and patient assessed outcomes:

After the treatment of one month the symptoms like nasal blockage, sneezing, rhinorrhea were slightly reduced after the first sitting. On the second course of treatment the intensity of the epistaxis was reduced to 12 episodes. After that, for a period of 11 months, the patient had only 6 episodes of epistaxis in entire treatment period. Then patient had 2 episodes of epistaxis for two months. Patient had no bleeding at the end of the treatment course. All the vitals and routine blood investigations were normal throughout the assessment period. The patient was closely observed for 1 year 4 months and he had no aggravation of symptoms. There were no adverse reactions/events observed during the entire course of treatment. Table 2 (see PDF) and Table 3 (see PDF) depict the Blood investigations before and after treatment. Figure 2 (see PDF) and 3 (see PDF) details the histopathology impressions and Figure 4 (see PDF) gives a CT impression of Para Nasal Sinus.

## Intervention adherence and tolerability:

Drug compliance form was maintained to ensure intervention adherence.

## Adverse and unanticipated events:

No adverse and unanticipated events occurred.

## Discussion:

Inflammatory sinonasal polyps are classified into main five types which are edematous, glandular, fibrous, cystic and angiomatous polyp [22]. Studies state that only angiomatous antrochoanal polyps present with epistaxis [23]. Given that, the primary clinical manifestation in this case is epistaxis, it is possible to classify this as an angiomatous variation since all polyps are described as inflammatory polyp in histological examinations [5]. In recent studies, the term "angiomatous polyp" has been suggested as being more suitable and compatible with the pathological features of extensive vascular proliferation, hemorrhage and infarction [24]. Here in this case, HPE also shows presence of large areas of hemorrhage which supports the confirmation of diagnosis as an angiomatous polyp. FESS plays the vital role in the management of antrochoanal polyps. Contraindications for FESS include Extra-ocular muscle injury, persistent diplopia, nasolacrimal duct injury, orbital hemorrhage or hematoma, orbital foreign body, optic nerve injury, blindness, sub periosteal abscess, abscess of the orbital tissue, orbital cellulitis, cavernous sinus thrombosis, enophthalmos, injuries to the vascular and nervous structures of the orbit [25, 26] and orbital emphysema leading to blindness [27].

So the pursuit of alternative medicine is increasingly significant. Although other conventional therapies for nasal polyps have been found to be effective, [28, 29-30] they have not met the minimal follow-up period of 6 months [31] since polyps show the higher recurrence rate. However, in this case the recurrence of ACP after FESS; relapse time was four years after initial surgery and two months after second. After undergoing Siddha treatment, patient experienced no symptoms of ACP over the period of one year and 4 months. Even though it seems to be a good prognosis follow up duration needs to be extended to confirm the effect. While discussing the possible mode of action of prescribed medicines; Herbo mineral Siddha formulation VKM contains Cinnabar and Borax along with other herbal ingredients in equal ratio. Modern research suggests that cinnabar helps in inhibition of the excessive release of inflammatory mediators [32]. In a fever rat model, Tang *et al.* reported that realgar up-regulated stress proteins such as HSP70 and HO-1, enhancing beneficial stress responses in the body. It also curbed the excessive release of inflammatory mediators like interleukin-1β, thereby mitigating pathological inflammation and preventing an inflammatory cytokine storm. These effects supported toxin elimination and protected key organs from damage [33].

Adathodai manappagu is in which Justicia adathoda plays the vital role which has an anti-inflammatory action over the respiratory tract. Ambroxol, a widely used secretolytic agent developed from vasicine, was shown to inhibit IgE-dependent mediator secretion from human MCs and basophils-the principal effectors of allergic inflammation. As compared to vasicine, ambroxol showed a greater effect in lowering basophil IL-4 and IL-13 secretions. It was also reported to reduce IgE-dependent p38 mitogen-activated protein kinase (MAPK) phosphorylation in basophils [34]. *Seenthil chooranam* is in which Tinospora cordifolia the major component plays the important role. It is having Immuno stimulant pharmaco-therapeutic activity. It's extract, a plant derived immunostimulant, significantly decreased (p < 0.00001) symptoms of allergic rhinitis like sneezing, nasal discharge, nasal obstruction and nasal pruritus [35]. Moreover, modern safety and pharmacological studies have proven the safety profile of *Seenthil chooranam* for long term use [36]. Oldenlandia umbellata the main component of *Impooral ilagam* is well-known in Siddha Medicine for its styptic property. It is also a drug that can be administered for bronchial asthma, as a decoction of the entire plant, a decoction made from its root and liquorice in the ratio 10:4 or as a powdered root given either with water or honey. A decoction of the root is also used as a febrifuge. Both leaves and roots are also deemed good expectorants and are recommended for treatment of asthma, bronchitis and bronchial catarrh [37]. *Muthu parpam* is also having anti-inflammatory activity and demonstrates that it is free from heavy metals, making it preferable for prolonged usage [38]. En bloc, these internal medicines are having an anti-inflammatory activity which might have helped in treating the antrochoanal polyposis. Follow up and non-recurrence should be evaluated further, even though there was substantial reduction in clinical features. Since it is a rare phenomenon there is no proper study conducted on antrochoanal polyposis cases with interventions in Siddha. So, this study paves a way to development of a treatment strategy which can be implemented in antrochoanal polyposis with long duration that is not responding to conventional treatment adopted by Allopathic system of medicine.

## Conclusion:

This case report shows that antrochoanal polyp can be successfully managed through Siddha regimen. In this case although the patient had repetitive recurrence of polyp and chronic symptoms even after repeated Surgeries, he treated with Siddha medications effectively and didn't have recurrence till date. This study can be useful in future references.

## Patient perspectives:

The patient self-reported that he was highly satisfied with the treatment as he had considerable reduction in symptoms. His quality of life was improved and he does not have recurrence of bleeding.

## Informed consent:

Written informed consent was obtained from the patient. The patient has given his consent for his images and other clinical information to be reported in the journal. The patient understand that name and initials will not be published and due efforts will be made to conceal identity, but anonymity cannot be guaranteed.

## Limitations:

Since this case report is based on single case which shows positive outcome this cannot be generalized. Further suitable clinical trials need to be conducted to assess efficacy. The recurrence in this condition persists over time, necessitating prolonged follow-up with the patient. End line CT scan couldn't be done since the patient was concerned about repeated exposure.
